# Metabonomic analysis of liver tissue from BALB/c mice with d-galactosamine/lipopolysaccharide-induced acute hepatic failure

**DOI:** 10.1186/1471-230X-13-73

**Published:** 2013-04-29

**Authors:** Bo Feng, Shengming Wu, Feng Liu, Yan Gao, Fangting Dong, Lai Wei

**Affiliations:** 1 Hepatology Institute, Peking University People’s Hospital, No.11 Xizhimen South Street, Beijing, 100044, PR China; 2 National Center of Biomedical Analysis, No.27 Taiping Road, Beijing, 100039, PR China

**Keywords:** Acute liver failure, Metabonomics, GC/MS, Liver tissue

## Abstract

**Background:**

Compared with biofluids, target tissues and organs more directly reflect the pathophysiological state of a disease process. In this study, a D-galactosamine (GalN) / lipopolysaccharide (LPS)-induced mouse model was constructed to investigate metabonomics of liver tissue and directly characterize metabolic changes in acute liver failure (ALF).

**Methods:**

After pretreatment of liver tissue, gas chromatography coupled to time-of-flight mass spectrometry (GC/TOFMS) was used to separate and identify the liver metabolites. Partial least squares – discriminant analysis models were constructed to separate the ALF and control groups and to find those compounds whose liver levels differed significantly between the two groups.

**Results:**

Distinct clustering was observed between the ALF and control mice. Fifty-eight endogenous metabolites were identified. Compared with the control mice, many metabolites, including sugars, amino acids, fatty acids, and organic acids, underwent significant changes in the ALF group, some of which differed from changes observed in plasma. Significant reduction of some important intermediate metabolites indicates that production of ketone bodies, the tricarboxylic acid and urea cycles, gluconeogenesis, glycolysis and pentose phosphate pathways are inhibited after GalN/LPS administration.

**Conclusions:**

GC/TOFMS can be a powerful technique to perform metabonomic studies of liver tissue. GalN/LPS treatment can severely disturb substance metabolism in the liver, with different effects on metabolites compared with those observed in the plasma.

## Background

Acute liver failure (ALF, sometimes referred to as fulminant hepatic failure) is a severe liver disease characterized by encephalopathy (International Normalized Ratio ≥ 1.5) and coagulopathy (any degree of altered mentation) in patients with previously normal liver function. It has a duration of less than 26 weeks and high mortality. Among ALF patients, those whose encephalopathy occurs within 7 days of onset of jaundice are named as hyperacute liver failure [1]. There are many causes of ALF, which vary in different countries. Currently, ALF related to drugs (including acetaminophen) accounts for more than 50% of all cases in some western societies, and hepatitis B virus (HBV) is still a relevant trigger, which is responsible for approximately 7% of cases in the US and 10% in Germany [2,3]. In contrast, viral hepatitis, especially hepatitis B, is the most important cause in China. However, following the application of antiviral drugs including various nucleoside analogues, hepatitis B-related ALF has decreased and drug-induced ALF has gradually increased in China.

The liver, as a center of substance and energy metabolism, undertakes principal synthesis, decomposition, excretion, transformation and other metabolic processes. Moreover, some of the enzymes and functions are liver-specific. These metabolic processes are likely to vary following liver diseases, and in different types of liver dysfunction, the variation may be different. ALF almost certainly results in significant changes of liver metabolites because of its rapid and severe liver cell necrosis; however, metabolic profiling of changes induced by ALF have not been well characterized. As an important analytical technology, metabonomics has been increasingly applied to various liver diseases, such as nonalcoholic fatty liver disease [4], liver cirrhosis [5], and hepatocellular carcinoma [6,7]. In previous studies, we performed plasma metabonomic analysis in BALB/c mice with fulminant hepatic failure induced by treatment with D-galactosamine (GalN) / lipopolysaccharide (LPS) [8,9]. Among 45 metabolites identified, some showed significant differences in plasma in response to GalN/LPS. However, assessment of liver tissue metabolites is of great value in metabonomic studies because it can provide more direct information on metabolism compared with assessment of biofluids.

In the current study, we performed metabonomic analysis of liver tissue in the GalN/LPS mouse model with the aim of directly characterizing changes of metabolism in ALF.

## Methods

### Animals

Male BALB/c mice (n = 24, 18–22 g) were purchased from the Academy of Military Medical Sciences (Beijing, China) and housed in a standard animal laboratory with a 12 h light–dark cycle. They were provided with water and standard mouse chow *ad libitum* and randomly divided into GalN/LPS-induced ALF (n = 10) and control (n = 10) groups. The current studies were carried out in accordance with the Chinese National Research Council guidelines and approved by the Subcommittee on Research Animal Care and Laboratory Animal Resources of the Peking University People’s Hospital.

### Establishment of ALF model and collection of liver tissue

The ALF model was established as described previously, with slight modification [8]. Approximately 6 h after GalN/LPS or saline treatment, the mice were sacrificed and the liver was immediately perfused through the left ventricle with chilled saline containing 25 U/mL heparin. Liver tissue was harvested in tubes and immediately snap-frozen in liquid nitrogen. After 2 h, the liver samples were transferred to −80°C and stored at this temperature until analysis. Serum biochemistry and liver histopathology were used to assess liver injury as previously reported [8].

### Pretreatment of liver tissue

A screw-cap vial was filled at least half full with 0.5 mm zirconia-silica beads. Liver tissue (200 mg) and extraction solvents (a mixture of chloroform, acetonitrile and water (1:2:1, v/v/v) were then added, ensuring that the microtube was filled almost to the top. Liver samples were homogenized using aMini-BeadBeater-16 (Biospec Co., USA) for 5 min and the vials cooled in ice-water for 1 min, then 20 μL of a ribitol stock solution (0.2 mg/mL in H_2_O) was added as an internal standard. The mixture was placed on a shaker at 70°C for 15 min and centrifuged at 10,000 × g for 10 min. The supernatant was separated, transferred into a GC vial, and then evaporated to dryness under a stream of N_2_ gas.

Metabolites in liver samples were derivatized prior to gas chromatography coupled to time-of-flight mass spectrometry (GC/TOFMS) analysis. Methoxyamine hydrochloride (20 μL, 20 mg/mL pyridine) was added to the dried fraction above, and continuously shaken at 30°C for 90 min.40 μL of N-methyl-N-trimethylsilyltrifluoroacetamide (MSTFA) with 1% TMCS was added and incubated at 37°C for 30 min. It was then kept at room temperature for 120 min before injection.

All chemicals were purchased from Sigma-Aldrich Chemical Co., Steinheim, Germany.

### GC-TOFMS analysis

The GC/TOFMS system consisted of an HP 6890 gas chromatograph and a time-of-flight mass spectrometer (Waters Co., Milford, MA, USA). Derivatized solutions (0.3 μL) were injected into a 30 m DB-5 column (250 μm i.d., 0.25 μm film; Agilent Technologies, Palo Alto, CA, USA) with 1 mL/min of helium as the carrier gas at a split ratio of 25:1. The temperatures of the injection, interface, and ion source were set at230°C, 290°C and 220°C, respectively. To achieve optimal separation, a gradient temperature program was set. After a 5-min solvent delay time at 70°C, the oven temperature was increased to 310°C in increments of 5°C/min, followed by a 1 min isocratic cool down to 70°C and an additional 5-min delay. MassLynx software (Waters Co.) was used to acquire the chromatographs. To identify the metabolites, NIST02 libraries with electron impact (EI) spectra were searched rigorously for all the peaks detected with the total ion current (TIC). Compounds were also identified by comparison of their mass spectra and retention times with those of commercially available references.

### Data processing and pattern recognition

Each sample was represented by a GC/TOFMS TIC chromatograph. Ribitol was added as an internal standard to correct minor variations during sample preparation and analysis. The relative intensity of each metabolite was normalized and expressed as 100 times the ratio of its peak area to that of ribitol on the same chromatograph.

The raw GC/TOFMS data were processed using the MarkerLynx applications manager software (Waters Co.), which incorporates a peak deconvolution package that allows the detection and retention-time alignment of the peaks eluting in each data file. MarkerLynx extracts components using mass chromatograms and lists the detected peaks according to their masses and retention times, together with their associated intensities. All the output data were exported from MarkerLynx to SIMCA-P plus (Umetrics, Sweden) for partial least squares discriminant analysis (PLS-DA) combined with orthogonal signal correction (OSC) to construct mathematical boundaries around each class and thereby maximize the separation between classes.

### Statistical analysis

Values are presented as mean ± SD. Comparisons of the measured metabolite intensities of the control and ALF groups were made using the unpaired Student’s *t* test with two-tailed distribution. *P*-values < 0.05 were considered statistically significant.

## Results

### Manifestations of the GalN/LPS-treated mouse model

Preliminary experiments showed that 80-90% of the mice were dead by approximately 6.5 h after GalN/LPS treatment because of the development of ALF. Compared with mice in the control group, GalN/LPS-treated mice showed slow movement and reactions, scattered hair, and very tumescent livers 6 h after treatment. With respect to liver morphology, GalN/LPS-treated mice presented with severe liver congestion, inflammation and massive necrosis as previously reported [8].

### GC/TOFMS TIC chromatogram of liver tissues

**Figure 1:**
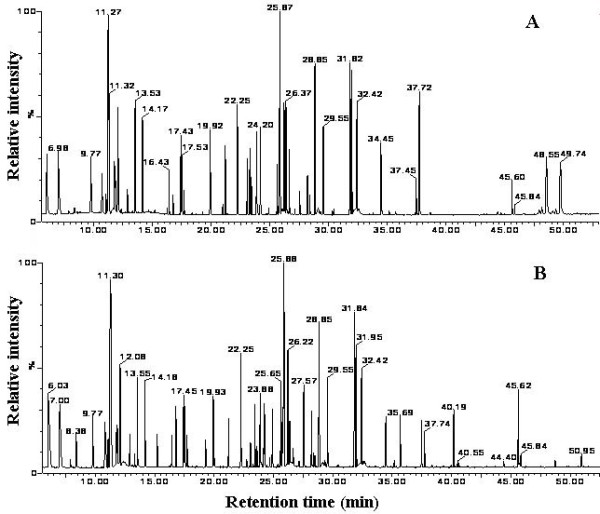
**Comparison of GC/TOFMS total ion current (TIC) chromatographs of liver tissue from mice in the control (A) and ALF (B) groups.** Each peak represents a metabolite, and the figures above the peaks represent their retention times.

The GC/TOFMS TIC chromatograms of liver samples from the saline control and GalN/LPS treatment groups are shown in Figure 1. The horizontal axis represents the time at which metabolites occur, and the vertical axis abundance. Each peak corresponds to a compound, and the figures above them represent their retention times. The area under a peak represents the relative abundance of the metabolite. Significant differences between the TIC profiles of the control and treatment groups were observed (Figure 1).

### Differences in scores plots between the control and ALF groups

**Figure 2:**
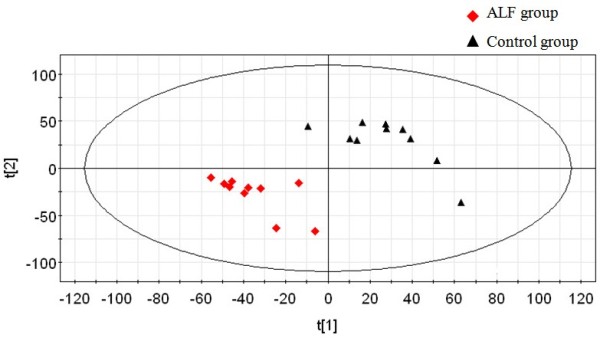
**Scores plot for PLS-DA of GC/TOFMS data derived from liver tissue of control and ALF mice.** The scores plot shows distinct clustering of the control and ALF groups. Triangle and diamond shapes represent control and ALF model groups, respectively.

To explore metabolic differences of liver tissues between the ALF model and the control group, the GC/TOFMS data were analyzed using multivariate statistics. The PLS-DA model (R^2^Y = 0.996, Q^2^Y = 0.72) shows clear separation between model and control samples (Figure 2). The first and second components accounted for 18.2% and 15.5% of the variance, respectively.

### Identification of endogenous metabolites in liver tissue

Two hundred and forty-three peaks were detected from GC/TOFMS TIC chromatographs of liver tissue. Based on the comparison with NIST02 libraries and commercially available reference compounds, 58 of the peaks were confirmed to be endogenous metabolites, such as sugars, amino acids, fatty acids, and organic acids. For further analysis, the metabolites were divided into an amino acid group and a non-amino acid group (Tables 1 and 2).

**Table 1:** Comparison of liver amino acid levels between the control and ALF groups

**No.**	**Retention Time**	**Amino Acids**	**Control Group**	**ALF Group**	**Ratio**^**c**^	***P***
1	6.98	alanine	197.63 ± 25.91	162.54 ± 23.76	0.82	0.0889
2	9.87	valine	81.52 ± 9.32	79.34 ± 13.53	0.97	0.8230
3	11.78	isoleucine	41.67 ± 8.16	43.00 ± 5.56	1.03	0.8026
4	11.86	5-oxy-proline	44.91 ± 11.28	50.17 ± 6.79	1.12	0.5499
5	12.10	glycine	157.18 ± 18.62	120.83 ± 11.80	0.77	0.0208
6	13.53	serine	98.42 ± 7.32	100.49 ± 9.06	1.02	0.7926
7	14.15	threonine	88.84 ± 10.42	77.97 ± 7.22	0.88	0.1624
8	15.20	B-alanine	38.87 ± 9.79	19.36 ± 1.63	0.50	0.0182
9	17.43	proline	99.95 ± 17.81	74.37 ± 7.51	0.74	0.0472
10	17.53	aspartate	54.20 ± 11.68	50.40 ± 4.85	0.93	0.6024
11	19.90	glutamate	98.41 ± 15.86	77.18 ± 5.01	0.78	0.0491
12	20.98	asparagine	2.06 ± 0.81	2.60 ± 0.78	1.26	0.5246
13	24.18	ornithine	41.78 ± 10.86	67.31 ± 14.79	1.61	0.1087
14	25.57	tyrosine	67.81 ± 9.84	32.34 ± 9.02	0.48	0.0008
15	26.37	lysine	10.61 ± 1.61	64.29 ± 12.27	6.06	0.0008
		TAA^a^	1123.87 ± 115.19	1022.21 ± 113.20	0.91	0.2207
		Gluconeogenic amino acids^b^	1003.66 ± 118.93	858.26 ± 85.36	0.86	0.0656

^a^ Total amino acids. ^b^Gluconeogenic amino acids include aspartate, serine, glycine, threonine, alanine, proline, lysine, tyrosine, valine, and isoleucine. ^c^Means ratio of metabolite levels between ALF and control groups.

**Table 2:** Comparison of non-amino acid metabolite levels between the control and ALF groups

**No.**	**Retention Time**	**Metabolites**	**Control Group**	**ALF Group**	**Ratio**^**a**^	***P***
16	6.02	lactate	238.84 ± 36.26	233.85 ± 38.91	0.98	0.8318
17	7.88	ethanedioic acid	5.92 ± 2.28	4.06 ± 1.51	0.69	0.1749
18	8.39	β-hydroxybutyrate	17.43 ± 1.96	5.09 ± 1.95	0.29	0.0017
19	10.91	urea	43.17 ± 6.07	20.54 ± 2.04	0.48	0.0013
20	11.20	phosphate	61.90 ± 10.34	38.67 ± 8.61	0.62	0.0279
21	11.30	glycerol	502.25 ± 42.61	459.58 ± 41.69	0.92	0.1886
22	12.35	succinic acid	30.58 ± 2.72	3.76 ± 1.68	0.12	0.0000
23	12.90	uracil	26.65 ± 5.82	29.78 ± 8.73	1.12	0.6393
24	13.31	fumaric acid	12.96 ± 3.60	11.67 ± 4.08	0.90	0.6120
25	13.78	pyruvate acid	8.46 ± 1.62	8.75 ± 0.96	1.03	0.7939
26	16.76	malic acid	45.24 ± 8.33	48.72 ± 4.17	1.08	0.4257
27	17.70	4-amino-butanoic acid	29.14 ± 3.50	33.28 ± 9.01	1.14	0.4762
28	18.50	erythronic acid	3.06 ± 0.77	1.01 ± 0.32	0.33	0.0064
29	19.28	2-piperidinedicarboxylic acid	19.73 ± 6.01	13.73 ± 5.56	0.70	0.0985
30	21.20	2,3,4-trihydroxy-butanal	99.30 ± 10.48	52.41 ± 12.11	0.53	0.0021
31	23.07	phosphoglyceride	114.73 ± 29.17	31.67 ± 9.72	0.28	0.0014
32	23.43	2-(bisamino)ethyl-phosphoric acid	48.32 ± 7.80	44.76 ± 9.20	0.93	0.3720
33	23.86	hypoxanthine	96.71 ± 15.64	62.57 ± 7.83	0.65	0.0425
34	24.26	arabinonic acid	1.66 ± 0.59	36.94 ± 5.53	22.25	0.0003
35	24.90	tetradecoic acid	74.92 ± 7.62	49.23 ± 8.42	0.66	0.0058
36	25.33	fructose	5.97 ± 1.30	3.19 ± 0.85	0.53	0.0092
37	25.63	glucose	439.00 ± 67.12	360.72 ± 42.74	0.82	0.0450
38	25.72	mannose	64.66 ± 14.56	37.53 ± 12.48	0.58	0.0137
39	26.22	galactose	158.06 ± 41.44	123.44 ± 19.37	0.78	0.0703
40	27.13	gluconic acid	66.74 ± 12.23	11.59 ± 4.73	0.17	0.0002
41	27.55	glucopyranose	98.84 ± 37.07	73.62 ± 27.60	0.74	0.3248
42	27.69	pantothenic acid	2.61 ± 1.12	2.47 ± 1.36	0.95	0.8489
43	28.18	purine	72.29 ± 18.01	35.07 ± 5.22	0.49	0.0180
44	28.48	6-deoxy-mannose	28.08 ± 7.66	10.19 ± 2.39	0.36	0.0052
45	28.84	palmitic acid	154.73 ± 16.61	162.99 ± 6.64	1.05	0.4927
46	29.54	inositol	85.87 ± 1.51	83.94 ± 6.01	0.98	0.5510
47	30.44	sedoheptulose	20.10 ± 7.80	3.07 ± 1.40	0.15	0.0121
48	31.82	linoleic acid	155.45 ± 17.62	144.69 ± 10.84	0.93	0.3358
49	31.93	oleic acid	116.51 ± 15.37	105.46 ± 7.70	0.91	0.3304
50	32.40	stearic acid	79.60 ± 5.93	83.70 ± 2.35	1.05	0.2288
51	33.63	glucopyranose phosphate	4.34 ± 1.76	2.36 ± 0.77	0.54	0.0538
52	32.44	arachidonic acid	62.30 ± 9.64	41.11 ± 8.21	0.66	0.0103
53	35.07	inositol monophosphate	5.50 ± 0.76	2.66 ± 0.70	0.48	0.0030
54	37.31	fructose phosphate	13.23 ± 4.61	0.50 ± 0.20	0.04	0.0061
55	37.44	ducosahexenoic acid	37.45 ± 5.03	37.42 ± 14.06	1.00	0.9963
56	37.70	adenosine	197.33 ± 36.90	29.66 ± 9.87	0.15	0.0008
57	40.15	maltose	41.62 ± 11.28	80.62 ± 27.90	1.94	0.0390
58	45.60	cholesterol	66.99 ± 11.90	35.54 ± 8.76	0.53	0.0330

^a^Means ratio of metabolite levels between ALF and control groups.

### Changes of metabolite levels after GalN/LPS treatment

The GC/TOFMS chromatograms showed changes in liver amino acid levels after GalN/LPS treatment. Fifteen amino acids were identified. Compared with the control group, levels of five amino acids (glycine, B-alanine, proline, glutamate and tyrosine) decreased and only lysine increased significantly in the ALF group. Levels of the other amino acids, as well as levels of total and gluconeogenic amino acids, were not different between the two groups (Table 1).

Forty-three non-amino acid metabolites were identified. Compared with those of the control group, levels of 22 metabolites decreased, 20 metabolites remained unchanged, and only one metabolite increased in liver tissues of mice from the ALF group. Among the eight sugars identified, fructose, glucose, mannose, 6-deoxy-mannose and sedoheptulose decreased, and only maltose was elevated. Lipids identified included saturated and unsaturated fatty acids, and some lipoids. Among saturated fatty acids, the level of tetradecanoic acid decreased, while palmitic acid and stearic acid showed no difference due to treatment. Among unsaturated fatty acids, the level of arachidonic acid decreased, while the remainder, including linoleic acid, oleic acid and docosahexaenoic acid, showed no differences between the two groups. Among lipoids, levels of phosphoglyceride, glucopyranose phosphate, fructose phosphate and cholesterol decreased significantly in the ALF group. Among other metabolites, β-hydroxybutyrate, urea, phosphate, succinic acid, erythronic acid, 2,3,4-trihydroxy-butanal, hypoxanthine, arabinonic acid, gluconic acid, purine, inositol monophosphate and adenosine also decreased significantly in the ALF group.

## Discussion

As one analytical technology for systems biology, metabonomics has displayed great power in screening metabolic biomarkers, describing metabolic pathways, and interpreting the function of complex biological systems [10]. Both biofluids and tissues can be used as samples for metabonomic analysis. Biofluids are used more frequently because they can be obtained more easily and in a relatively non-invasive manner. However, the majority of substances in biofluids are easily affected. For example, because blood is a transport organ, exogenous materials are transported to various organs, and endogenous substances are exchanged between different organs by plasma. Lesions of each organ may change levels of plasma metabolites and thus affect plasma metabonomics. Therefore, in the case of a disease, the target tissue or organ may be more important than biofluids for metabonomic analysis because it can directly reflect pathophysiological responses to the disease process [11].

Based on the GC/TOFMS chromatograms, it was observed that GalN/LPS treatment induced significant changes of some metabolite levels in the liver. In total, compared with those of the control group, levels of 27 metabolites decreased, 29 metabolites did not change, and only two metabolites increased in liver tissues of mice in the ALF group.

Among the amino acids identified, liver levels of glycine, B-alanine, proline, glutamate and tyrosine decreased and lysine increased significantly in the ALF group. There were no differences in the total levels of amino acids between the two groups. In the case of sugars, the levels of fructose, glucose, mannose, 6-deoxy-mannose and sedoheptulose all decreased while maltose was elevated. Among fatty acids, tetradecanoic acid and arachidonic acid decreased. Most lipoids including phosphoglyceride, glucopyranose phosphate, fructose phosphate and cholesterol decreased significantly in the ALF group. Many other metabolites, such as β-hydroxybutyrate, urea, phosphate, and succinic acid, decreased significantly in the GalN/LPS-induced ALF group.

Changes in the liver levels of many metabolites were inconsistent with their levels in plasma. In our previous study, significantly elevated levels of almost all of the identified amino acids were observed in GalN/LPS-treated mice compared with those in the control group. In contrast, in the current study several metabolites (including phosphate, phosphoglyceride, fumaric acid and malic acid) that previously showed elevated plasma levels either decreased, or did not change, in liver tissue. Furthermore, urea and glucose, which showed no change in plasma, decreased in the liver tissue of the ALF group. It is well known that plasma metabonomics reflects systemic metabolic effects associated with GalN/LPS stimuli, while tissue-targeted metabonomic analyses enable more precise investigation of local metabolism. ALF is a severe liver disorder with devastating consequences and it encompasses a pathophysiological response associated with rapid deterioration of liver functions. Its pathology presents as severe hepatocyte inflammation, massive apoptosis and/or necrosis [12]. A multitude of metabolites inside liver cells are released into the blood within a short time, which impacts liver levels of metabolites.

Massive apoptosis and/or necrosis cause deterioration of liver function. At the same time, the majority of amino acids, sugars, and fatty acids (among other metabolites) decrease significantly in liver tissues in ALF. The consequent lower levels of many metabolites inside liver cells thus fail to provide sufficient raw materials for further biosynthesis and energy metabolism. An absolute reduction of β-hydroxybutyrate, succinic acid and urea, and a relative reduction of fumaric acid and malic acid, indicate that the production of ketone bodies, tricarboxylic acid and urea cycles are significantly inhibited during ALF.A significant decrease of fructose phosphate illustrates that the gluconeogenesis, glycolysis and pentose phosphate pathways may also be disturbed. The significant decrease of liver levels of hypoxanthine, purine and adenosine suggest that exposure to GalN/LPS disrupts nucleotide metabolism. These disturbed pathways are related to several mechanisms of GalN/LPS injury to the liver. Oxidative stress is induced by release and accumulation of many reactive oxygen species in response to GalN/LPS. Reactive oxygen species are fatal to liver cells and result in cell death [13]. Significantly decreased levels of the polyunsaturated fatty acid arachidonic acid show evidence of lipid peroxidation [14]. Finally, GalN depletes the uridine triphosphate pool and thus inhibits mRNA and protein synthesis [15].

## Conclusions

GC/TOFMS combined with multivariable statistics can be used to perform metabonomics analysis of liver samples. Compared with control mice, significant differences in the liver levels of many metabolites were found in the ALF mouse model, and some of these changes in liver differed from those observed in plasma. Distinct clustering was observed between the ALF and control mice. Significant reductions of some important intermediate metabolites indicate that production of ketone bodies, the tricarboxylic acid and urea cycles, gluconeogenesis, glycolysis and pentose phosphate pathways are inhibited after GalN/LPS administration. Liver-targeted metabonomic analyses are able to provide relatively direct and precise information regarding localized changes in metabolism in ALF.

## Abbreviations

ALF: Acute liver failure; GalN: d-galactosamine; LPS: Lipopolysaccharide; GC/TOFMS: Gas chromatography/time-of-flight mass spectrometry; TIC: Total ion current; OSC: Orthogonal signal correction; PLS-DA: Partial least squares discriminant analysis.

## Competing interests

All authors declare that they have no competing interests.

## Authors’ contributions

BF contributed to the experimental design, experimental process, data acquisition, statistical analysis, and drafted the manuscript. SW contributed to the experimental process, data acquisition, statistical analysis, and drafted the manuscript. FL participated in study planning and statistical analysis. YG participated in construction of animal models and planning. FD contributed to study design, data analysis, and drafting the manuscript. LW participated in design, funding analysis, and manuscript drafting. All authors read and approved the final manuscript.

## Pre-publication history

The pre-publication history for this paper can be accessed here:

http://www.biomedcentral.com/1471-230X/13/73/prepub
